# Evaluating Emotional Response and Effort in Nautical Simulation Training Using Noninvasive Methods

**DOI:** 10.3390/s25175508

**Published:** 2025-09-04

**Authors:** Dejan Žagar

**Affiliations:** Faculty of Maritime Studies and Transport, University of Ljubljana, 1000 Ljubljana, Slovenia; dejan.zagar@fpp.edu

**Keywords:** effort, emotions, noninvasive sensors, human factor error

## Abstract

The purpose of the study is to research emotional labor and cognitive effort in radar-based collision avoidance tasks within a nautical simulator. By assessing participants’ emotional responses and mental strain, the research aimed to identify negative emotional states associated with a lack of experience, which, in the worst-case scenario, could contribute to navigational incidents. Fifteen participants engaged in multiple sessions simulating typical maritime conditions and navigation challenges. Emotional and cognitive effort were evaluated using three primary methods: heart rate monitoring, a Likert-scale questionnaire, and real-time facial expression recognition software. Heart rate data provided physiological indicators of stress, while the questionnaire and facial expressions captured subjective perceptions of difficulty and emotional strain. By correlating the measurements, the study aimed to uncover emotional patterns linked to task difficulty with insight into engagement, attention, and blink rate levels during the simulation, revealing how a lack of experience contributes to negative emotions and human factor errors. The understanding of the emotional labor and effort in maritime navigation training contributes to strategies for reducing incident risk through improved simulation training practices.

## 1. Introduction

The maritime industry is inherently demanding, with Officers of the Watch often facing extended periods of navigation that lead to significant physical and mental fatigue [1]. The integration of advanced navigational instruments on ship bridges, while enhancing operational capabilities, introduces additional cognitive load [2], which contributes to maritime incidents. Decision-making and situational awareness, as highlighted by the European Maritime Safety Agency (EMSA), affect human actions and are implicated in a substantial proportion of maritime accidents, suggesting the need to address the psychological challenges faced by Officers of the Watch (OOW) [3].

The present study aims to identify negative emotional states associated with a lack of navigational experience, which, in the worst-case scenario, can contribute to maritime incidents. The findings are intended, in the long term, to support the integration of standard marine training programs with targeted strategies for emotional resilience and stress management to promote a balanced reliance on technology, ensuring that it serves to enhance human judgment rather than replace it [4]. By examining emotional patterns associated with task difficulty and the limitations of navigation technology, we seek to identify patterns that may suggest changes in emotional labor and effort. Emotional stress, exacerbated by long working hours, isolation, environmental unpredictability, and the high stakes of navigation, can impair critical cognitive functions such as situational awareness, attention, and problem-solving [5]. Advancements in technology, while designed to improve safety, can inadvertently lead to diminished manual navigation skills, particularly during high-stress scenarios in which over-reliance on automated systems may serve as a coping mechanism for operators who, overwhelmed by environmental and operational pressures, bypass critical decision-making processes [6,7]. Thus, addressing both the psychological and technological aspects of maritime safety is imperative. By enhancing emotional resilience and promoting effective stress management strategies among seafarers, alongside the prudent use of navigational technology, the long-term goal is to reduce human factors in marine accidents, approaching not only effective learning processes in simulators but also safer navigation practices of maritime professionals. Thus, simulator-based training has been recognized as an effective method for preparing Officers of the Watch for high-risk, complex environments such as a ship’s bridge or an aviation cockpit. In maritime contexts, research has shown that stressors such as time pressure, system malfunctions, and environmental challenges can significantly influence cognitive load, situational awareness, and decision-making performance [8,9]. Emotional states, including anxiety and overconfidence, have been identified as contributors to human error during tasks [10]. Researchers have typically examined the role of emotional and psychological responses during simulator training, demonstrating that participants’ stress responses may influence task prioritization and highlighting that cognitive workload and emotional strain interact with performance and error likelihood [11,12].

As mentioned, the learning process in a simulator often causes strain and fatigue, which affects the ability to retain information [13,14]. Similarly, long navigation itself causes strain and fatigue, and the use of modern navigational instruments on ship bridges adds additional cognitive load. This is assumed to be a major factor in maritime incidents [15], which are frequently linked to emotional and psychological challenges faced by Officers of the Watch (OOW). The research is an early-stage, exploratory study based on a small sample of maritime students without professional sea experience. The findings should therefore be interpreted as preliminary and indicative, serving as a basis for future studies involving professional mariners. Thus, this exploratory study seeks to contribute to addressing the mentioned vulnerabilities by providing preliminary insights that may inform a multipronged approach in future research, integrating standard marine training programs with a focus on emotional resilience [9,16], with the goal of uncovering emotional patterns associated with task difficulty. The novelty of the study lies in its application of a multimodal, non-invasive measurement approach—combining physiological measurements, self-assessment, and facial expression analysis —within a controlled radar-based collision avoidance simulation. While similar research often focuses on experienced mariners for potential real-life insight [8,12,17], this study examines emotional labor and cognitive effort in inexperienced maritime students to identify and quantify early-stage stress patterns linked to navigational challenges [10,11]. By correlating sensor data, this research provides a novel perspective on emotional resilience in complex radar-based navigation scenarios.

## 2. Materials and Methods

The primary goal of this study was to assess emotions and effort in relation to the experience required for radar-based collision avoidance tasks within a simulated maritime environment. The focus was on understanding how emotional demands and effort evolved as participants engaged in repeated sessions, gaining experience and refining their skills. To achieve this, the study employed a combination of physiological, subjective, and behavioral measures to evaluate emotional responses and cognitive effort during the simulation tasks.

### 2.1. Participants

Participants were recruited from the Automatic Radar Plotting Aids (ARPA) navigation class at the Faculty of Maritime Studies and Transport in Portorož. The study included 12 male and 3 female participants (N = 15) with an average age of 21.1 years (SD = 3). None of the participants had prior experience in maritime navigation, ensuring that the study could effectively assess the impact of training on individuals new to the field. Before the session, participants were informed about the task, goal, and data collection to ensure their safety throughout the research. They were also advised that they could request additional information at any time. It was explicitly explained that they had the right to withdraw their consent at any point without needing to provide justification, and they were assured that withdrawing from the study would not impact any other services they might be receiving. To confirm their understanding and agreement, participants provided their signatures on a consent document. Ethical considerations were reviewed and confirmed by the Commission for Research and Development Activities (KRRD) of the Faculty of Maritime Studies and Transport at the University of Ljubljana. The research was approved during the commission’s 7th correspondence session on 5 June 2024, which confirmed that the study adhered to all ethical standards, including compliance with the General Data Protection Regulation (GDPR) and alignment with standard research practices. The experimental design aligned with established learning practices and methodologies, offering insights into the emotional demands and effort involved in maritime navigation training.

### 2.2. Instrumentation

**Figure 1:**
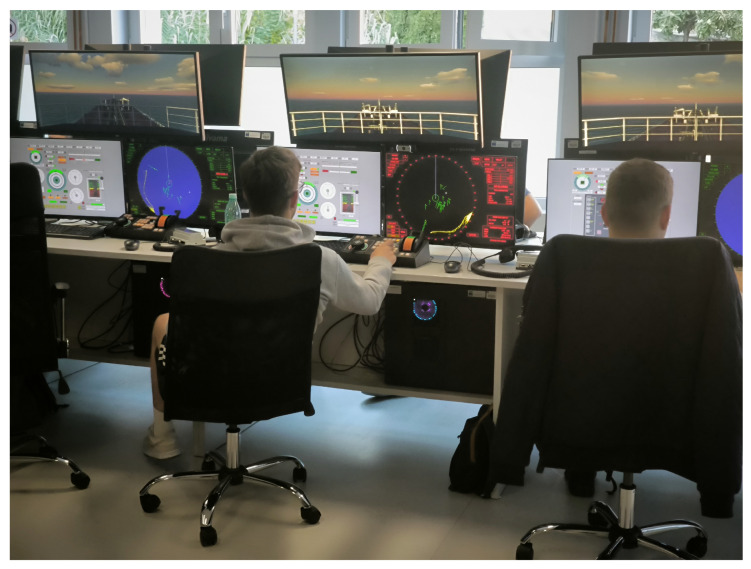
Nautical simulation environment. Participants perform collision avoidance and maneuvering.

The research was conducted in a nautical simulator (Wärtsilä, Portsmouth, UK) featuring a modern, ergonomic console with standard navigation tools, including multifunctional displays, a conning station, radar, and an overhead monitor for simulating an external view (Figure 1). For the realistic nautical simulation environment, Wärtsilä NTPRO 6000 software was used [18], which supports Automatic Radar Plotting Aids training [19]. The operational challenges were similar to those in real maritime settings, such as collision avoidance, maneuvering through dense traffic, and maintaining situational awareness.

The second type of instrumentation combined objective measurements with self-reported feedback to assess participants’ strain and effort during the simulation tasks. Heart rate values were recorded immediately before and after each session using participants’ personal smartwatches, enabling the detection of changes that could indicate increased arousal, stress, or effort. In addition, participants completed a post-session Likert-scale questionnaire [20] to capture their perceptions of task difficulty, workload, and overall experience. The survey questions (Table 1), developed in line with studies by [21,22], enabled participants to express their emotional and cognitive experiences in a structured way, offering subjective insights into how they felt during each task. Likert-scale responses [23] from 1 to 5 allowed for a quantifiable analysis of strain and effort, providing data on how participants felt during the simulation. The questionnaire provided subjective, self-reported insights into participants’ experiences during the training sessions; therefore, the results should be interpreted with caution. Although such data are vulnerable to response bias, including the influence of expectations, the survey outcomes were used to provide contextual understanding and to complement heart rate and facial expression data rather than to serve as standalone evidence.

**Figure 2:**
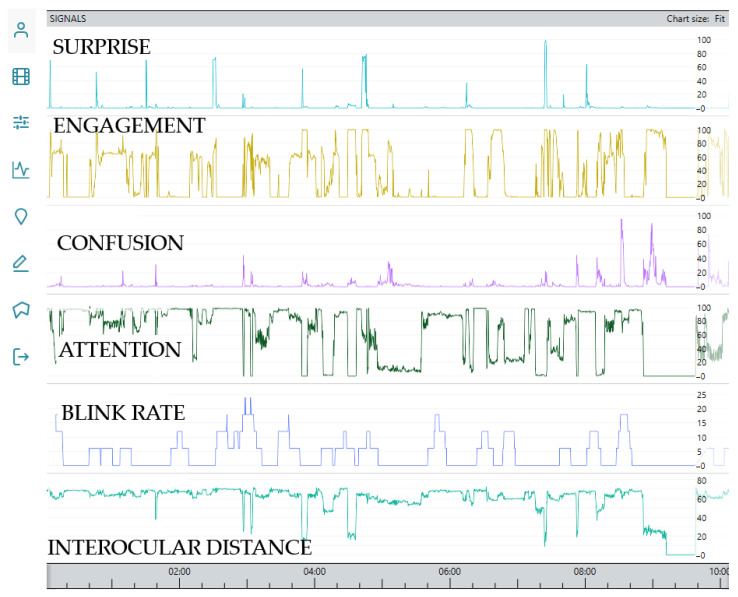
Analysis of facial expressions: the x-axis presents time in minutes, and the y-axis presents norm values. The captured video signal was analyzed at a 30 Hz sampling rate, where subtle facial expressions and micro-movements were detected, reflecting features such as surprise, engagement, confusion, attention, blink rate, and interocular distance.

The third type of measurement tool employed in this study involved the analysis of facial expressions using computer software, offering an objective approach to assessing participants’ emotional states during the simulation tasks [24]. The software (iMotions ver. 10.0), based on video signal analysis, captured participants’ facial movements with a 30 Hz sampling rate while they performed collision avoidance scenarios, measuring emotional reaction features. Facial expression analysis integrates the Affectiva Affdex facial coding module, which is a validated tool widely used in academic and applied research for the automated detection of facial movements and emotion-related expressions [25]. The module measures facial muscle activity by tracking distances and relative movements between predefined facial landmarks, and the changes are continuously analyzed to detect subtle variations in expression and gaze. Together, the features provide emotional responses during the simulated training scenarios. In addition to heart rate and the Likert scale questionnaire, the included emotions were surprise, engagement, confusion, and attention levels. Blink rate and inter-ocular distance were also extracted as secondary indicators of cognitive effort and focus. The facial video was recorded using an HD-PRO webcam positioned in front of the participant [26]. It is acknowledged that lower frame rates, low resolution, and blurry images can reduce the temporal precision and accuracy of facial expression detection, particularly for brief or subtle expressions (Figure 2). During post hoc analysis, we identified microexpressions that typically reveal emotions, which people may not consciously register.

Despite the fact that students generally do not have issues completing anonymous surveys, as demonstrated in our case, in which all participants responded without withdrawing, we encountered an interesting challenge. Since they had the right to withdraw their consent at any time without providing a justification, the video recordings occasionally excluded several participants. For ethical reasons, we did not specifically investigate the reasons or any potential gender differences; however, a few students declined video recording in all sessions, while others participated in just over half of the sessions on average. Nevertheless, in combination with heart rate monitoring and self-reported Likert-scale data, the facial expressions provided an additional view of the emotional labor and effort analysis, enabling a more nuanced understanding of the emotional dynamics as participants gained experience in the simulation, allowing for the identification of subtle emotional cues that may not be captured by physiological or self-reported data alone. Throughout the sessions, we enhanced the evaluation of how emotions and effort evolve in response to increasing familiarity and competence in handling complex navigational tasks.

### 2.3. Experimental Protocol

As mentioned, sessions were incorporated into the participants’ regular academic program and designed in accordance with the Standards of training [27]. The simulations aimed to replicate real-world maritime conditions and navigation challenges, creating a realistic learning environment that allowed for skill development while enabling the assessment of emotional labor and effort. Before the data collection phase, participants, according to their school schedule, underwent a three-week familiarization period to become accustomed to the simulator equipment, including radar buttons, ARPA radar operation, collision avoidance procedures, and trial maneuvers. This ensured that all participants had a baseline understanding of the tools and concepts necessary for the study. Prior to each session, participants were briefed on the task, research objectives, and data collection procedures to ensure their safety and comprehension of the study’s purpose. Following the familiarization phase, participants took part in four weeks of data collection, which encompassed simulation exercises, surveys, and video recordings for post hoc analysis. Each of the six sessions typically consisted of four recorded simulation scenarios, ensuring a comprehensive dataset for further evaluation. Each session focused on radar-based collision avoidance tasks, requiring participants to make real-time navigational decisions to prevent potential collisions. These tasks were structured to evaluate the participants’ ability to process information and respond under simulated operational pressures. The scenarios remained consistent across sessions, ensuring comparability of results over time. At the beginning of each session, participants received a short briefing detailing their simulated location, such as the North Adriatic, Port of Koper, the English Channel, the approach to Göteborg, and the approach to New York. They were informed about the type of vessel they were navigating; whether it was a container ship, bulk carrier, or tanker; and the specific navigational context, such as operating within a traffic separation zone or a narrow channel, or maneuvering during a port approach.

We could not avoid the weaknesses of the experimental protocol, particularly the potential variability in individual learning curves and prior experience [28,29]. Despite the three-week familiarization phase, some participants had different levels of competence in radar operation and collision avoidance, which could have influenced their emotional and cognitive responses during the simulation [30,31]. While the scenarios remained consistent across sessions, individual decision-making and situational awareness can introduce variability that may impact the measurement of emotional labor and effort. Some participants became more comfortable with the simulator and tasks over time, leading to reduced effort not necessarily due to skill improvement but simply due to familiarity with the system. This could make it difficult to distinguish between actual learning gains and a decline in perceived task difficulty due to repeated exposure, as revealed by analysis of participants’ microexpressions. The study also relies on self-reported survey data to assess emotions and effort, introducing the risk of subjective bias [32]. Participants, when comparing with emotion analysis, might underreport stress or effort due to social desirability bias, or their responses may fluctuate based on external factors such as fatigue or mood on a given day, which also affect heart rate measurements before and after the sessions. These limitations were considered when interpreting the results related to emotional labor and effort in nautical simulation training.

### 2.4. Data Analysis

The data analysis employed a repeated-measures statistical approach combined with descriptive statistics, pre–post analysis, and outlier handling to assess trends in facial expressions, heart rate, and questionnaire responses over multiple simulation sessions [33]. The mean values for heart rate and each questionnaire item were computed to determine central tendencies, providing insight into overall participant experience. The box-and-whisker method [34,35] was used in the visualization to illustrate the spread of data, ensuring that outliers were visually distinguished from the main distribution. This approach allows the evaluation of individual- and group-level trends over time to examine changes in physiological and self-reported measures across simulation sessions. Repeated-measures analysis reveals changes in variables such as heart rate and questionnaire responses over multiple sessions. The use of central tendency measures (mean method) and dispersion metrics (standard deviation, interquartile range, and whiskers in boxplots) helps quantify overall trends and individual variations [34]. Additionally, by segmenting data into first-session vs. last-session comparison (groupby method), we applied a pre–post analysis framework, assessing differences in emotional labor and effort between the beginning and end of the session. Outlier detection and removal followed a statistical approach using the interquartile range (IQR) method, ensuring that extreme values did not distort central tendency measures [35].

The video signal from a standard web camera was analyzed using iMotions analysis software, version 10.0 [24], which enabled the detection of subtle facial expressions and micromovements, yielding a range of extracted features, including indicators of surprise, engagement, confusion, attention, blink rate, and interocular distance. The initial step involved visualizing features over time, allowing for a direct temporal interpretation of facial dynamics throughout the session. The extracted features were exported as .csv files and subjected to further statistical comparisons using Python, Spyder, ver.5 code, which were conducted between data from the first and last sessions to assess changes in facial expressions over the course of training. Mann-Whitney U correlation analysis [36] was performed to identify relationships between different facial metrics. The test statistic is given by

(1) $$U=n_{1}n_{2}+\frac{n_{1}\left(n_{1}+1\right)}{2}-R_{1}$$

where $n_{1},n_{2}$ are sample sizes and $R_{1}$ is the rank sum of sample 1. Bootstrapping is used to estimate confidence intervals by resampling with replacement. With the mentioned nonparametric method, we examined patterns of engagement and attentional focus to determine whether participants exhibited reduced effort or emotional labor as they progressed. Statistically significant correlations between facial features and performance metrics provide insight into improvements in task efficiency and confidence.

## 3. Results

Despite the comparatively small sample size of the sessions (N = 60), the responses are based on a substantial number of observations, ensuring a relatively robust dataset for analysis. This helps reduce the impact of outliers and individual variations, providing more reliable insights into participants’ experiences over multiple simulation sessions. A summary of heart rate values before and after the simulation is provided in Table 2, which presents the statistical measures: mean, standard deviation (std), and percentiles (50th, 75th, and 25th percentiles), representing the range of values. The HR before the session has a mean of 74.83 bpm, indicating the average heart rate of all participants before starting the simulation. The HR after the session has a slightly higher mean of 76.18 bpm, suggesting that, on average, participants experienced a small increase in heart rate following the simulation. The standard deviation (std) for HR before and HR after shows variability in heart rate responses across participants. The 50th percentile (median) represents the middle value of the data, where half of the participants have a higher HR and half have a lower HR. The 75th percentile shows the point below which 75% of the values lie, indicating the upper end of the central range of responses, while the 25th percentile represents the lower end of the central range, where 25% of participants had lower heart rate values. This describes the spread of heart rate data, offering a view of how heart rate changed before and after the simulation as well as how much variation there was across participants.

**Figure 3:**
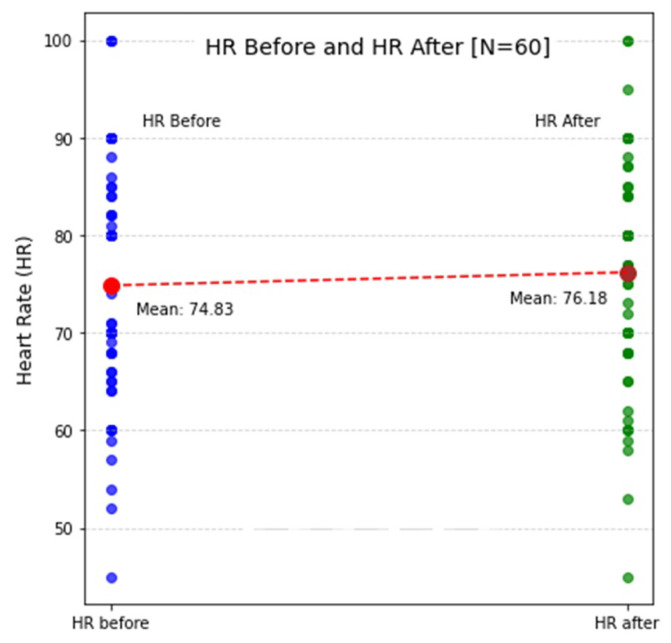
Comparison of mean HR before and HR after reveals changes in heart rate across sessions, indicating an increase in physiological activation in response to the simulation.

Similarly, Figure 3 visualizes heart rate measurements before and after each simulation session, providing insight into physiological responses over time. The y-axis represents HR values, while the x-axis corresponds to before and after sessions. The height of the blue dots indicates the HR value before the simulation, while green dots represent HR after. A red dot marks the mean HR value across groups, offering a reference point for overall trends. The upward slope of the red trend suggests that HR after the session is generally higher than HR before, indicating an increase in physiological activation or stress response due to the simulation [37].

This pattern may reflect heightened cognitive and emotional engagement during the session, leading to elevated heart rates, and the distribution of green dots demonstrates individual variability in HR responses, indicating habituation or differing stress tolerance levels. The overall statistical summary for the questionnaire responses is presented in Table 3, displaying the average values for all participants. By grouping the data, the table provides insight into the simulation experience, including the average time taken to complete the survey, highlighting differences in both physiological responses and subjective perceptions and offering a comprehensive view of how participants experienced the simulation sessions.

The mean values for the questionnaire responses (Table 1 and Table 3) indicate general trends in participants’ perceptions. The question about difficulty (Q3) was rated at a moderate level, suggesting that participants found the tasks somewhat challenging but not overly difficult, with responses varying moderately across individuals. Stress (Q4) had the lowest average rating, indicating that most participants did not experience or recognize high levels of stress during the simulation, and responses remained relatively consistent. Certainty (Q5) had the highest rating, showing that participants generally felt confident in their responses, with a relatively low standard deviation of 0.87, suggesting minimal variation among participants. Effort (Q6) was reported at a moderate level, indicating that the workload required to complete the tasks was neither minimal nor excessive, though responses varied to some extent. Success (Q7) was rated above the midpoint, suggesting that participants generally perceived their performance as fairly successful; however, with a standard deviation of 1.17, success ratings showed the highest variability, reflecting differences in individual expectations, prior experience, or adaptation to the simulation over time.

**Figure 4:**
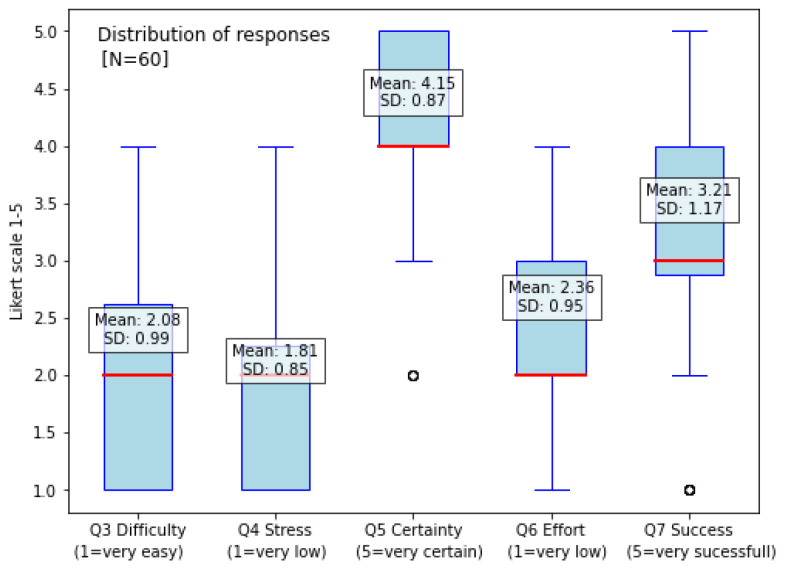
Distribution of responses. The figure shows the distribution of responses and the mean values. Results indicate changes in HR across sessions, reflecting a response to the simulation.

The distribution of responses for questions Q3 to Q7 are presented in Figure 4, which summarizes the variability and central tendency of participants’ self-reported experiences. Each box corresponds to a specific question: Q3 (difficulty), Q4 (stress), Q5 (certainty), Q6 (effort), and Q7 (success). The whiskers extend to show the full range of responses, while the boxes represent the interquartile range (IQR), covering the middle 50% of values. The red median line within each box highlights the central tendency of responses, while any points beyond the whiskers indicate outliers, reflecting individual differences in perception. The lower values for difficulty (Q3), stress (Q4), and effort (Q6) indicate lower perceived challenge, stress, and mental effort, respectively. In contrast, higher values for certainty (Q5) and success (Q7) represent greater confidence and perceived success.

The survey responses in Table 4 reveal patterns in self-reported measures. Negative values indicate a decrease in the last session compared to the first session, while positive values indicate an increase in the last session compared to the first session. Difficulty ratings generally decrease over time, reflecting increased familiarity with the task and improved competence. Stress levels tend to decline, reinforcing the idea that repeated exposure helps participants manage anxiety and uncertainty more effectively. Certainty in performance shows a positive trend, highlighting the role of experience in building confidence. Effort, while expected to decrease, exhibits variability: some participants develop more efficient strategies, reducing their perceived workload, whereas others continue to exert high levels of concentration. Reported success tends to improve, aligning with the expectation that training leads to better performance outcomes. By analyzing the statistical differences, we gain an understanding of how they adapt to the simulation over time. When comparing heart rate before the session in the first attempt, we observe that five participants showed a decrease, while two out of fifteen remained unchanged. By the last session, six participants exhibited a decrease, and three showed no change. The general downward trend suggests reduced emotional labor and effort as participants became more accustomed to the process. However, heart rate after the session often remained elevated or even increased, indicating that while participants may feel more prepared at the beginning, the task continues to require a level of engagement that maintains physiological activation [38]. Outliers, such as participant seven, suggest potential false HR readings, while participant six’s unchanged or inconsistent responses may indicate a lack of engagement with the survey questions.

The results in Table 5 indicate the means of first and last session, indicating that participants became faster in completing the survey over time, with an average reduction of 85.93 s in the last session compared to the first, suggesting that participants had increased familiarity with the survey and possibly reduced cognitive load in recalling their experiences. However, the heart rate before the simulation increased by 6 bpm, which was less expected. This increase could be partially explained by the presence of outliers in the data. Some participants arrived late for the session and had to run to arrive in class on time, temporarily elevating their heart rate before the simulation began. These elevated readings may have skewed the HR-before results, making it appear as though participants were experiencing increased presimulation stress when, in reality, some were simply experiencing the physiological effects of rushing to class. When the outliers were removed, the increase in HR before fell to 0.67 bpm (Table 6), which better reflects the actual emotional and cognitive anticipation of the simulation rather than incidental physical exertion, indicating heightened anticipation, increased awareness of the complexity of the tasks, or emotional investment in performance.

Participants rated the difficulty of the simulation 1.19 points higher in the last session, which reflects a deeper understanding of task intricacies rather than an actual decline in skill. Similarly, stress levels increased by 0.93 points, which could be linked to a greater awareness of the consequences of errors, higher expectations of their own performance, and evolving cognitive and emotional engagement with the simulation. These findings align with the decline in certainty (−0.27 points) about task completion, suggesting that as tasks became harder, confidence wavered despite increased experience. The required effort increased by 0.33 points, reinforcing the idea that over time, more demanding tasks were introduced. Even though participants became more skilled, effort remained high. However, despite challenges, the reported success increased by 0.57 points, showing that participants ultimately felt more accomplished in their performance. An elevated heart rate after a simulation session can be attributed to physiological stress responses, where the autonomic nervous system remains activated even after the session concludes, as well as anxiety [39]. The physiological response, in which HR after the session increased by 3.6 bpm (without outliers: 3.83 bpm), suggests a different kind of stress response, indicating that while participants managed to complete the tasks, the intensity or pressure of the session left a lasting physiological effect, aligning with the concepts of emotional labor and effort, where cognitive and emotional engagement remains high even as proficiency improves.

### Facial Analysis

We used a similar approach in the final part of the analysis of facial micro-movements, which includes features such as surprise, engagement, confusion, attention, blink rate, and interocular distance. Despite students generally having no issues completing anonymous surveys, as shown by the full participation of all 15 students (Figure 3), an interesting challenge emerged with video recordings. Since participants had the right to withdraw their consent at any time without providing a justification, video capture was declined by several participants. The specific reasons behind these decisions and potential differences based on gender were not examined due to ethical considerations. A total of 38 recordings were captured across all sessions, averaging 6.3 recordings per session (Table 7). Three students declined video recording entirely, while most consented to video recording in at least half of the sessions. Two participants consented to video recording in five sessions, and three participated in only two sessions or fewer.

A statistical comparison of the mean value between the first and last session provides insight into potential trends and changes in participants’ emotional labor and effort responses over time (Table 8). Surprise showed a slight increase in mean value from 1.95 to 2.14, with a marginal rise in standard deviation, suggesting no substantial change in variability. Engagement exhibited a minor increase in mean from 22.87 to 23.92, indicating a slight broadening in response variation. Confusion levels increased from a mean of 3.39 to 3.92, hinting at a slight uptick in uncertainty or cognitive load. Attention showed a notable decrease, with the mean dropping from 62.92 to 54.65, indicating a possible decline in sustained focus over sessions. Blink rate decreased from a mean of 6.22 to 4.57, suggesting a change in eye activity as sessions progressed. Interocular distance also decreased significantly, from a mean of 54.99 to 40.89, indicating changes in head positioning and movement patterns over time.

The paired *t*-tests indicate that none of the features show statistically significant differences between the first and last sessions. Surprise (t = −0.35, *p* = 0.73), engagement (t = −0.41, *p* = 0.69), and confusion (t = −0.44, *p* = 0.67) all exhibit *p*-values well above 0.05, suggesting no meaningful changes. Attention (t = 0.62, *p* = 0.54) and blink rate (t = 0.65, *p* = 0.52) also do not show significant variation. Interocular distance presents the most notable difference (t = 1.76, *p* = 0.089), but the *p*-value remains above 0.05, indicating only a marginal trend toward significance.

While some mean differences are observed, assumed to be due to the small sample size, we may not have been able to detect a statistically significant difference. However, we also cannot exclude the possibility that these differences were due to random variation rather than systematic changes between sessions.

Thus, because the normal distribution of the differences between groups could not be assumed, we used the Mann–Whitney U test [36,40]. The results in Table 9 indicate that, except for blink rate and interocular distance, features do not show statistically significant differences between the first and last session.

Surprise, engagement, attention, and confusion show no significant differences, with *p*-values above 0.05, indicating that they do not vary meaningfully between groups. The blink rate *p*-value is near 0.05, suggesting a promising trend toward significance, indicating sensitivity to changes in effort, even though the result is not statistically significant at the conventional threshold. Interocular distance is the only feature with a significant *p*-value (below 0.05), indicating a clear difference between groups. The effect sizes imply that while the differences are not statistically significant, they may still be practically meaningful, but the small sample size makes it harder to detect subtle effects.

## 4. Discussion

The differences between the first and last sessions for each participant suggest how their physiological and psychological responses evolve over time. The time required to complete the survey generally decreased, suggesting that participants became more familiar with the process and required less effort to answer the questions. Physiological features suggest that participants did not experience significant reductions in effort over time, indicating that even with repeated exposure to the simulation, physiological readiness and alertness remain slightly heightened. When comparing the overall trends across different modalities, both the heart rate data and the facial-expression results show a comparable increase in arousal-related variables over the sessions, even though they are based on different sample sizes. One possible explanation is that as participants progress, they become more aware of performance expectations, leading to a maintained or increased level of mental preparation before each session. The data indicate that the nature of the task demands sustained focus and preparedness, regardless of experience [41]. While participants became more confident in their approach, their responses to the simulation showed sustained effort and mental exertion, potentially due to increased situational awareness or a greater self-imposed performance standard as they progressed.

However, variability in responses suggests that not all participants follow the same trajectory; while some adapt quickly, others may struggle with certain aspects of the simulation, leading to inconsistencies in physiological and self-reported measures and providing insight into individual differences. Some participants consistently reported similar levels of difficulty, stress, effort, certainty, and success, while others displayed greater fluctuations across sessions. If certain individuals show increasing stress or effort despite repeated exposure, it may suggest difficulties in adaptation, fatigue, or variations in task complexity. Addressing outliers helped clarify the underlying trends and patterns, suggesting that while participants became more accustomed to the simulation process, the effort and emotional demands of the task remained substantial, requiring sustained engagement and effort throughout the training period. The results also suggest that while most emotional features remained stable, changes in blink rate and interocular distance could reflect shifts in participants’ visual behavior, potentially due to fatigue, adaptation, or effort levels over repeated sessions.

Physiologically, a lower blink rate is often associated with heightened focus and attention, particularly when performing tasks that require attention and engagement [42]. When a task becomes more challenging, participants may subconsciously reduce their blink rate as a response to the increased mental effort needed. The results, despite lacking enough statistical power to draw a strong conclusion, open a research direction that will be the focus of future studies. On the other hand, participants in the first session had a smaller interocular distance compared to the last session, which can be linked to physiological responses related to visual focus. Psychologically, this may be a response to situational awareness, where narrowing the gaze facilitates improved visual attention and perception of fine details [43]. The *p*-value for interocular distance is significant, indicating that participants adjusted their gaze and visual focus to cope with effort, attention, and engagement.

From a theoretical perspective, this study contributes by integrating multiple measurement approaches—objective heart rate monitoring, facial expression analysis, and subjective self-assessment—in a nautical simulator context. The findings offer preliminary insights into how a lack of navigational experience can influence emotional states and cognitive effort during navigational tasks, thus supporting models that link emotional labor and cognitive load to decision-making processes in demanding environments. From a practical perspective, the results indicate the potential value of incorporating emotional resilience and stress management components into maritime training programs. Even though the current findings are exploratory, the observed patterns suggest that simulation exercises can be designed to both challenge navigational skills and develop coping strategies for managing emotional strain. Additionally, the methodological framework tested here—particularly the integration of multimodal data collection—can inform the design of future training assessments and the use of real-time monitoring of crew readiness and performance.

## 5. Conclusions

From a broad perspective, this study supports efforts to reduce human error in maritime accidents, where integrating biometric monitoring into bridge resource management strategies could improve decision-making and crew performance. This paper presents preliminary findings that reveal patterns of potential relevance to maritime training; however, due to the limited sample size and methodological constraints, the results should be interpreted as a foundation for more extensive future studies. By correlating the data, the analysis offers a perspective on emotional resilience in complex radar-based navigation scenarios. All three modalities show a similar trend of increased arousal and effort over the simulation sessions, where the convergence of indicators suggests that early-stage stress responses can be identified and quantified even among inexperienced maritime students, providing valuable insight for the development of training strategies aimed at enhancing emotional resilience. The findings contribute to research on visual attention and cognitive load, where reductions in blink rate and interocular distance may serve as biomarkers of sustained cognitive effort, fatigue, or increased task familiarity. The emotional response and effort analysis focused on the differences between the first and last sessions of each participant to examine how their physiological and psychological responses evolved over time. The time to complete the survey decreased, indicating greater familiarity with the process and less effort. Heart rate showed a slight increase, suggesting that anticipatory stress and anxiety did not significantly decrease over time. The use of personal smartwatches in our experimental design offered advantages in terms of simplicity and widespread availability. However, in future research, we plan to include additional metrics, such as electrodermal activity and electrocardiography, to provide a more robust assessment. This is because despite efforts to minimize the influence of uncontrolled factors, the observed changes may still have been affected by such factors, which limits the reliability of heart rate alone as an indicator of physiological response. Despite increasing familiarity, the physiological readiness and alertness required before each session remain slightly heightened due to increased awareness of performance expectations, indicating that participants maintained anticipatory engagement or that self-imposed performance standards rise. Both blink rate and interocular distance reflect effort, with lower blink rate and reduced interocular distance signaling greater mental engagement.

In operational environments, future research can explore how officers manage cognitive and emotional demands under varying conditions. Real-time emotional labor and effort measurement would provide insights into fatigue, stress-related decision-making errors, and coping strategies. Incorporating physiological tracking in bridge operations can help identify cognitive overload early, enabling interventions such as optimized shift rotations or adaptive training to enhance safety.

## Figures and Tables

**Table 1:** The survey questions used to express emotional experiences. Note: Question 7 was included from the second session onward.

Question	Text	Likert Scale
Q3	How difficult did you find the simulation task?	1 = Very Easy, 5 = Very Difficult
Q4	How much stress did you experience during the task?	1 = No Stress, 5 = Very Stressful
Q5	How confident (certain) were you in your ability to handle the simulator?	1 = Completely Uncertain, 5 = Completely Confident
Q6	How much mental effort did you exert during the simulation?	1 = Very Little, 5 = Very Much
Q7	How do you perceive your performance compared to the previous session?	1 = No Improvement, 5 = Significant Improvement

**Table 2:** HR rate: The data show the mean values across all participants and sessions.

N = 60	HR Before	HR After
Mean	74.83	76.18
Std. dev.	12.23	11.43
25%	67.50	69.50
50%	72.50	78.50
75%	84.00	84.00

**Table 3:** Questionnaire responses: overall distribution of survey response statistics of all sessions.

N = 60	Time (s)	Difficulty (Q3)	Stress (Q4)	Certainty (Q5)	Effort (Q6)	Success (Q7)
Mean	96.28	2.08	1.81	4.15	2.36	3.21
Std. dev.	51.30	0.99	0.85	0.87	0.95	1.17

**Table 4:** First vs. last session: survey responses show individual patterns, with difficulty ratings generally decreasing over time, indicating growing familiarity and competence. Negative values indicate a decrease, while positive values indicate an increase in the response.

Participants	Time (s)	HR Before	Q3	Q4	Q5	Q6	Q7	HR After
1	−61	−17	0	2	0	0	0	12
2	−82	13	2	2	0	1	−2	−1
3	−6	−4	−1	0	1	−1	1	−11
4	−53	−5	2	1	0	1	0	0
5	−145	−9	0	0	0	0	0	−4
6	0	0	0	0	0	0	0	0
7	−57	45	1	2	−2	0	1	45
8	−133	−5	1.5	1	0	1	1.5	−5
9	−122	8	1	−2	0	0	2	−15
10	−242	20	0	0	0	−3	1	20
11	−145	32	3	1	−1	2	3	−8
12	−4	2	3	2	−2	1	0	5
13	−82	0	2	2	0	2	1	10
14	−121	4	2	1	0	0	-1	0
15	−36	6	1	2	0	1	1	6

**Table 5:** Means generally indicate increased familiarity with questions; however, the increase in “HR before” is less expected.

N = 15	Time (s)	HR Before	Q3	Q4	Q5	Q6	Q7	HR After
Mean	−85.93	6	1.17	0.93	−0.27	0.33	0.57	3.6
Std. Dev.	66.35	16.11	1.19	1.16	0.8	1.23	1.21	14.61

**Table 6:** Statistical comparison of the first vs. last session with removed outliers: “HR before” is significantly lower and better reflects the participants’ actual engagement.

N = 15	Time (s)	HR Before	Q3	Q4	Q5	Q6	Q7	HR After
Mean	−88.9	0.67	1.04	1.14	0.0	0.52	0.53	3.83
Std. Dev.	32.18	4.77	0.86	0.86	0.0	1.23	1.21	14.61

**Table 7:** Summary of participant consent data: although a total of 60 recordings were expected, only 38 were obtained, as several participants either declined or withdrew consent for video recording during one or more sessions.

Number of participants	15
Expected recordings	60
Actual recordings	38
Mean consent	3.17 (Participants consented to more than 3 sessions)
Standard Deviation	1.11
Decline	3 (Three students declined video recording entirely)

**Table 8:** Comparison of the emotional features between the first and last session.

Feature	Mean First	Std. First	Mean Last	Std. Last
Surprise	1.95	10.15	2.14	10.63
Engagement	22.87	35.22	23.92	36.34
Confusion	3.39	11.30	3.92	12.34
Attention	62.92	40.41	54.65	42.24
Blink-rate	6.22	7.57	4.57	6.82
Interocular distance	54.99	31.88	40.89	21.51

**Table 9:** Blink rate and interocular distance suggest potential significant differences.

Feature	*p*-Value	Bootstrap *p*-Value	Effect Size
Surprise	0.7045	0.6735	0.0959
Engagement	0.8071	0.5949	0.1237
Confusion	0.1719	0.1591	0.3287
Attention	0.7123	0.9260	−0.0217
Blink rate	0.0894	0.0557	−0.4481
Interocular distance	0.0051	0.0041	−0.6598

## Data Availability

The datasets used and/or analyzed during the current study are available from the corresponding author upon reasonable request.
